# Etanercept-induced sarcoidosis presenting with bilateral panuveitis: diagnostic value of ocular signs and therapeutic response to IL-17A inhibition—a case-based review

**DOI:** 10.1007/s10067-025-07759-4

**Published:** 2025-10-18

**Authors:** Tommaso Bonifazi, Alessio Cerquaglia, Barbara Iaccheri

**Affiliations:** Department of Ophthalmology, University of Perugia: Hospital S. Maria Della Misericordia, Perugia, 06156 Italy

**Keywords:** Drug-induced sarcoidosis, Etanercept, Paradoxical adverse events, Psoriasis, Secukinumab, TNF-alpha inhibitors

## Abstract

**Background:**

Tumor necrosis factor-alpha (TNF-α) inhibitors are effective treatments for autoimmune diseases but may cause paradoxical adverse events (PAEs), including sarcoid-like granulomatosis. Etanercept, a soluble TNF receptor fusion protein, has been more frequently associated with this phenomenon compared to monoclonal antibodies.

**Case presentation:**

We describe a 61-year-old man with chronic plaque psoriasis on long-term etanercept therapy who developed bilateral visual symptoms. Ophthalmologic evaluation revealed granulomatous panuveitis with retinal vasculitis and macular edema. Imaging and lung biopsy confirmed pulmonary sarcoidosis with non-caseating granulomas. Etanercept was discontinued, and systemic corticosteroids were started. Because of steroid dependence, secukinumab (an IL-17A inhibitor) was introduced, together with an intravitreal dexamethasone implant for refractory macular edema. Over a 4-year follow-up, the patient achieved complete and sustained resolution of ocular inflammation with stable systemic control, without the need for further corticosteroids.

**Discussion:**

This case illustrates a rare but clinically significant presentation of etanercept-induced sarcoidosis with primary ocular involvement. Unlike typical psoriatic uveitis, the bilateral granulomatous pattern prompted further systemic work-up, which revealed otherwise silent pulmonary disease. To our knowledge, this is the first long-term report of etanercept-induced ocular sarcoidosis successfully managed with secukinumab, which allowed steroid withdrawal. While IL-17A inhibitors are not yet approved for uveitis, available data and our observation suggest a potential role in selected cases.

**Conclusion:**

Etanercept-induced ocular sarcoidosis is a rare but clinically significant PAE. Prompt recognition and multidisciplinary management are crucial to prevent vision loss. IL-17A inhibition may represent a promising steroid-sparing therapeutic option when TNF-α inhibitors are contraindicated or cause paradoxical reactions.

## Introduction

Tumor necrosis factor-alpha (TNF-α) inhibitors are a cornerstone in the management of chronic immune-mediated diseases, including rheumatoid arthritis, ankylosing spondylitis, psoriatic arthritis, plaque psoriasis [1], and, in selected cases, refractory sarcoidosis [2]. This therapeutic class comprises monoclonal antibodies (infliximab, adalimumab, golimumab), a PEGylated Fab fragment (certolizumab pegol), and the soluble TNF receptor fusion protein etanercept [1].

Despite their efficacy, TNF-α inhibitors may paradoxically trigger conditions they are otherwise used to treat—a phenomenon known as paradoxical adverse events (PAEs) [1, 3]. Among these, sarcoid-like granulomatosis is one of the best characterized, most often affecting the lungs and skin [4]. Epidemiological data suggest that the risk of drug-induced sarcoidosis-like reactions (DISR) varies across agents: etanercept has been disproportionately implicated compared to monoclonal antibodies, likely due to pharmacodynamic differences. Unlike infliximab or adalimumab, which neutralize both soluble and membrane-bound TNF-α, etanercept predominantly targets the soluble form, potentially leading to incomplete cytokine blockade and altered Th1 immune responses [5–7].

Although ocular involvement is rare, it carries major clinical relevance, as uveitis can be sight-threatening if unrecognized or undertreated [8, 9]. Awareness of this possibility is crucial, especially when the inflammatory phenotype diverges from that typically associated with the underlying disease.

Here, we describe a case of etanercept-induced sarcoidosis presenting with bilateral granulomatous panuveitis as the initial manifestation. The case is notable both for the sentinel role of ocular findings in revealing otherwise asymptomatic systemic disease and for the successful long-term management with secukinumab, an interleukin-17A (IL-17A) inhibitor, following corticosteroid dependence.

## Clinical features and interventions

A 61-year-old Italian man presented in November 2020 with progressive visual blurring, floaters, and pain in both eyes. His medical history included type 2 diabetes mellitus, mild hepatic steatosis, and chronic plaque psoriasis, which had been treated with etanercept for the past 6 years.

**Fig. 1:**
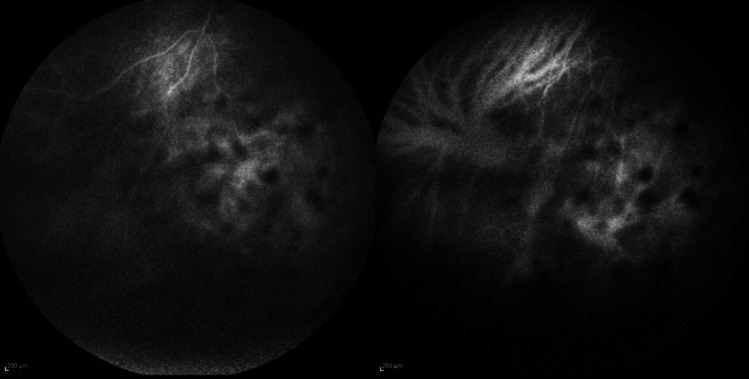
FAG and ICGA of the left eye showing blocked fluorescence due to clusters of inflammatory cells in the inferior sectors of the vitreous chamber (snowballs)

**Fig. 2:**
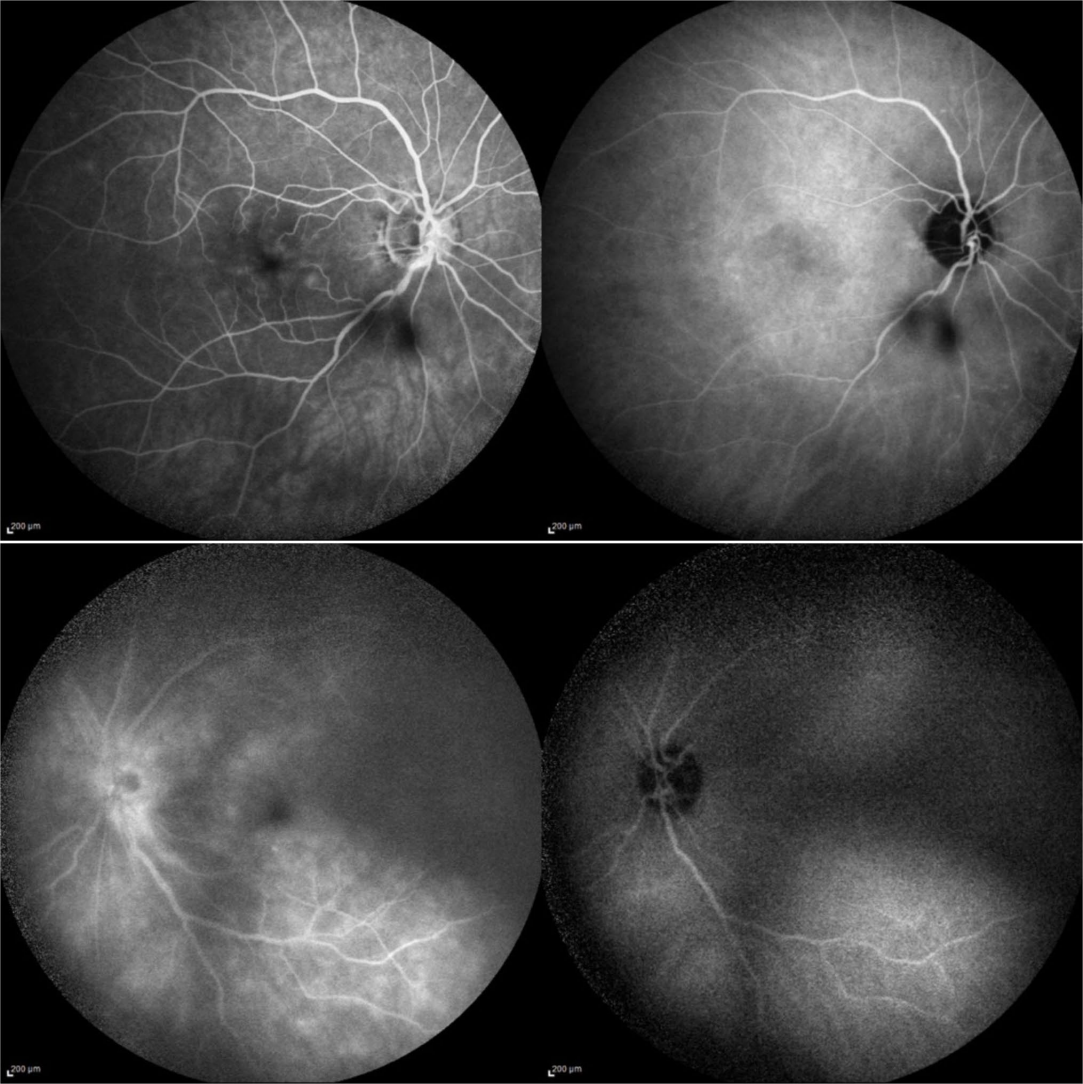
Artero-venous phases of FAG and ICGA showing mild dye leakage from retinal capillaries in the macular area and optic disc in both eyes; in the left eye (shown in the bottom images) peripapillary leakage can be seen. Visibility was not good in the left eye due to intense vitreous inflammation

Ophthalmologic examination revealed anterior chamber inflammation with tyndall 2 +, flare 2 +, and mutton-fat keratic precipitates, as well as posterior chamber inflammation characterized by vitritis 2 + and snowball opacities (Fig. 1). Best-corrected visual acuity (BCVA) was 20/20 in the right eye and 20/40 in the left eye. Fluorescein and indocyanine green angiography showed bilateral retinal capillaritis and optic disc leakage (Fig. 2). Spectral-domain optical coherence tomography (SD-OCT) revealed macular edema in the left eye. These findings supported the diagnosis of bilateral granulomatous panuveitis.

A broad differential diagnosis was considered. Infectious causes were systematically excluded by negative serological tests (*Bartonella* spp., *Borrelia*, hepatitis B and C viruses, syphilis, interferon-gamma release assay), and the inflammatory pattern was not consistent with psoriatic uveitis, which typically presents as anterior and unilateral disease. Laboratory results were otherwise unremarkable except for iron-deficiency anemia and mild leukocytosis. Erythrocyte sedimentation rate (ESR), C-reactive protein (CRP), and serum ACE were within the normal range.

High-resolution chest computed tomography (HRCT), performed during the systemic evaluation, unexpectedly revealed bilateral interstitial lung involvement with micronodules, together with bilateral hilar and mediastinal lymphadenopathy. Bronchoalveolar lavage (BAL) showed increased cellularity (415,400 cells/mL) and an elevated CD4/CD8 ratio (2.9). Transbronchial lung biopsy confirmed the presence of non-caseating granulomas.

A diagnosis of etanercept-induced sarcoidosis with ocular and pulmonary involvement was made. Etanercept was discontinued and systemic corticosteroids (prednisone 50 mg/day) were initiated with gradual tapering. This treatment led to a marked reduction of inflammation (from flare 2 +, tyndall 2 +, vitritis 2 + to low-grade flare + 1, tyndall 0, vitritis + 1, with corresponding visual improvement), but the patient remained steroid-dependent for several months with partial relapses. Three months after presentation, an intravitreal dexamethasone implant (Ozurdex®) was administered in the left eye for refractory macular edema.

Given the persistence of low-grade inflammation and steroid dependence, secukinumab (an IL-17A inhibitor) was introduced in April 2021. From that point onward, the patient achieved complete and sustained remission of ocular inflammation without further relapses or corticosteroid use. At follow-up in April 2025—4 years after starting secukinumab—BCVA was 20/20 in the right eye and 20/25 in the left, with no evidence of active uveitis. Dermatologic and systemic symptoms also remained stable without additional immunosuppression.

A chronological overview of ocular and systemic manifestations in relation to therapy is provided in Table 1.

**Table 1:** Clinical timeline of the case

Date/timepoint	Event/findings	Intervention/outcome
Nov 2020 (presentation)	Bilateral visual blurring, floaters, ocular pain. Clinical examination: granulomatous panuveitis (flare 2 +, tyndall 2 +, vitritis 2 +), BCVA 20/20 OD, 20/40 OS. SD-OCT: macular edema OS	Start diagnostic work-up. Infectious causes excluded
Dec 2020	HRCT: bilateral micronodules, hilar/mediastinal lymphadenopathy. BAL: increased cellularity, CD4/CD8 ratio 2.9. Lung biopsy: non-caseating granulomas	Diagnosis of etanercept-induced sarcoidosis. Discontinued etanercept. Systemic corticosteroids initiated (prednisone 50 mg/day, tapering)
Jan–Mar 2021	Partial improvement: inflammation reduced (flare + 1, vitritis + 1). Still steroid-dependent with relapses	Continued steroids
Feb 2021 (3 months)	Persistent macular edema OS	Intravitreal dexamethasone implant (Ozurdex®) OS
April 2021	Ongoing low-grade uveitis, steroid dependence	Initiated secukinumab (IL-17A inhibitor)
2021–2025	Complete remission of ocular inflammation. No relapses, no steroids needed	Maintained on secukinumab. Psoriasis and systemic symptoms controlled
Apr 2025 (latest FU)	BCVA 20/20 OD, 20/25 OS. No signs of active inflammation	Stable systemic and dermatologic status

## Discussion

TNF-α inhibitors have transformed the management of many chronic inflammatory diseases, yet they may be associated with paradoxical adverse events (PAEs), including sarcoid-like granulomatosis [1, 3]. Although uncommon, these reactions are increasingly recognized.

Epidemiological data suggest that the risk of drug-induced sarcoidosis-like reactions (DISR) differs among agents. In a comprehensive review of more than 100 cases, Miedema and Nunes reported that etanercept accounted for nearly half of all documented DISR, followed by adalimumab and infliximab, with most events arising after more than 2 years of treatment [8]. This disproportionate association is thought to reflect pharmacodynamic differences: while infliximab and adalimumab neutralize both soluble and membrane-bound TNF-α, etanercept primarily targets the soluble form, which may lead to incomplete cytokine blockade and altered Th1 regulation [4–7]. More recent evidence supports this pattern. A 2025 systematic review by Andolfi et al., analyzing nearly 300 published cases, again identified etanercept as the most frequently implicated TNF-α inhibitor, confirming its disproportionate association compared with monoclonal antibodies [10].

The concept of DISR provides a structured framework for interpreting such cases. This entity has been defined as a syndrome clinically and histologically indistinguishable from idiopathic sarcoidosis, but arising in temporal association with a specific treatment and typically regressing after its discontinuation [9]. Diagnostic evaluation generally requires three elements: compatible clinical or radiological features, histological evidence of non-caseating granulomas, and improvement following withdrawal of the suspected drug [8, 9]. Importantly, persistence of low-grade disease activity for several months after drug discontinuation has been described, reflecting the heterogeneity of DISR and indicating that delayed resolution remains compatible with a drug-related etiology [8].

Ocular involvement in DISR is rare and likely underrecognized [11, 12]. In their 2022 review, Sobolewska et al. emphasized that biologics, particularly TNF-α inhibitors, may induce sarcoid-like uveitis [13], although pulmonary and cutaneous manifestations are more common [11–13]. Rare case reports, such as that by Saifee et al., have even described severe late-onset ocular presentations with profound vision loss [14]. In our patient, the inflammatory phenotype was atypical: bilateral granulomatous panuveitis, rather than the unilateral anterior form usually observed in psoriatic uveitis [15]. This mismatch, together with negative infectious screening, prompted systemic evaluation, ultimately revealing pulmonary abnormalities and biopsy-proven sarcoidosis. Such findings underscore the sentinel role of ocular disease in uncovering otherwise silent systemic pathology.

From a therapeutic standpoint, discontinuation of the offending biologic remains the cornerstone of management [8, 9, 13]. Case reports have documented regression of sarcoidosis after stopping anti-TNF therapy with adjunctive corticosteroids [16, 17]. In our patient, systemic corticosteroids led to a substantial reduction in ocular inflammation (flare 2 +, tyndall 2 +, vitritis 2 + to flare + 1, tyndall 0, vitritis + 1, with visual improvement). However, low-grade inflammation persisted, the patient remained steroid-dependent, and relapse of macular edema required intravitreal dexamethasone (Ozurdex®) in the left eye. Similar persistence of low-grade activity for several months after drug withdrawal has been documented in DISR series [8], and our patient’s course reflects this heterogeneity, underscoring that delayed resolution remains fully compatible with a drug-related etiology.

Secukinumab was introduced in 2021 to address steroid dependence, in addition to controlling systemic psoriatic disease. Its efficacy in psoriasis and psoriatic arthritis is well established [18], while its role in uveitis is supported by more limited evidence. The rationale for IL-17A inhibition stems from its involvement in Th17-mediated inflammation and granuloma biology. Clinical data, though still preliminary, provide relevant context: Hueber et al. [19] demonstrated activity of secukinumab (AIN457) in psoriasis, rheumatoid arthritis, and uveitis in a proof-of-concept trial, while Letko et al. [20] confirmed efficacy and safety of intravenous secukinumab in non-infectious uveitis requiring steroid-sparing therapy, with superior outcomes observed for intravenous versus subcutaneous dosing. Although later phase III trials reported mixed results and secukinumab has not been approved for uveitis, these findings support the biological plausibility of IL-17A blockade in selected cases.

In our case, the introduction of secukinumab marked a decisive therapeutic turning point: the patient became steroid-independent and maintained complete remission of ocular inflammation. At 4 years of follow-up, no relapses have been observed, with preserved visual acuity and stable systemic and dermatologic control.

This report highlights several unique aspects. First, it describes an atypical clinical course in which bilateral panuveitis preceded the diagnosis of systemic sarcoidosis, underlining the diagnostic value of ophthalmic findings. Second, it details the management of etanercept-induced sarcoidosis complicated by ocular involvement, with challenges related to prolonged steroid dependence. Finally, it provides, to our knowledge, the first long-term description of secukinumab use in this context, suggesting that IL-17A inhibition may represent a promising therapeutic option when TNF-α inhibitors are contraindicated or elicit paradoxical reactions.

Taken together, these observations emphasize the importance of individualized therapeutic strategies, timely recognition of DISR, and further research into the immunological mechanisms and alternative targeted treatments.

## Conclusion

Etanercept-induced sarcoidosis is a rare paradoxical reaction that may initially manifest with ocular involvement, such as bilateral granulomatous uveitis. Prompt recognition and discontinuation of the offending agent are essential to prevent irreversible visual damage. This case emphasizes the sentinel role of ophthalmic findings in uncovering systemic disease and illustrates the potential of IL-17A inhibitors such as secukinumab as effective steroid-sparing options in patients who cannot continue TNF-α inhibitors. Further studies are warranted to clarify the long-term safety and efficacy of IL-17A blockade in non-infectious uveitis and drug-induced sarcoidosis-like reactions.

## Data Availability

All data generated or analyzed during this study are included in this published article.
